# Language Perception and Processing

**DOI:** 10.3390/brainsci16101063

**Published:** 2026-10-01

**Authors:** Ratree Wayland

**Affiliations:** Department of Linguistics, University of Florida, Gainesville, FL 32611-5454, USA; ratree@ufl.edu

When I proposed this Special Issue, I used the term language processing broadly to encompass not only the perception and processing of speech sounds but also the use of syntactic, semantic, and contextual information during language comprehension. The resulting collection reflects this breadth, with studies examining spoken and written language across several levels of linguistic processing.

This Special Issue includes 13 contributions. Twelve report empirical studies, and one presents a research protocol. Fishero et al. (Contribution 1), Oganyan et al. (Contribution 2), Zheng et al. (Contribution 3), and Venkateswaran and Wayland (Contribution 4) examine how acoustic cues, perceptual salience, and phonological structure influence speech perception and accentedness judgments. Fu et al. (Contribution 5), Leung et al. (Contribution 6), Liu et al. (Contribution 7), Tremblay and Kim (Contribution 8), and Hong et al. (Contribution 9) investigate how language experience, learning, cross-linguistic influence, and language dominance shape speech production and/or perception. Mathew et al. (Contribution 10) and Meng et al. (Contribution 11) use event-related potentials to examine morphosyntactic case processing and the processing of moral and semantic violations, respectively. Hromatko and Tadinac (Contribution 12) examine variation in verbal performance and hemispheric asymmetry associated with the menstrual-cycle phase, while Zuo et al. (Contribution 13) present a protocol for studying the neural processing and integration of phonological features in children with autism spectrum disorder.

Across these different topics, a common theme is that language processing depends on the selective use of available information. Which information becomes consequential depends on the acoustic and linguistic properties of the signal, the structure and context in which it occurs, the demands of the task, the listener’s language experience, and individual neurocognitive or biological characteristics. The contributions approach this selectivity using behavioral, acoustic, computational, eye-tracking, and electrophysiological methods across a range of languages and participant populations. The studies of speech perception show that listeners do not use all of the information available in the speech signal equally. For example, Fishero et al. (Contribution 1) found that differences in both fundamental frequency and speaking rate between target and masker speech could improve speech segregation when each was manipulated separately. When both cues varied, however, only the effect of fundamental frequency was reliable overall; speaking-rate differences contributed when the target and masker had the same fundamental frequency. Oganyan et al. (Contribution 2) found that listeners’ responses to novel consonant clusters were better predicted by the perceptual salience of the consonants than by their position in the sonority hierarchy. Zheng et al. (Contribution 3) showed that Sichuan Mandarin listeners were more sensitive to the/n–l/contrast before/i/than before/a/, corresponding to more robust contrast-relevant acoustic information in the/i/context. Venkateswaran and Wayland (Contribution 4) similarly found that not all phonological features that differed in production predicted listeners’ accentedness judgments. These findings show that the presence of acoustic or phonological differences does not guarantee that they will affect perception. Their contribution depends on their availability, salience, reliability, and relevance to the task.

The importance of linguistic structure is also evident in morphosyntactic processing. Mathew et al. (Contribution 10) found different ERP responses to ergative and nominative case violations in Hindi compound light verb constructions. Ergative case violations elicited a late positivity, whereas nominative case violations elicited a biphasic negativity and late positivity. These findings indicate that the neural response to a case violation depends on the structural configuration in which it occurs.

Building on previous ERP studies comparing moral or social-norm violations with semantic violations [1,2], Meng et al. (Contribution 11) directly compared moral and semantic violations while keeping the critical region constant and varying the preceding context. For example, “sped up” appeared in the same target sentence, “So she sped up the car immediately.” It constituted a moral violation when children were crossing the road, a semantic violation when the car had no gas, and a neutral continuation when the driver was running late. Moral violations elicited a sustained posterior late positive component, while semantic violations produced a later positivity with a descriptively more frontal distribution, although the difference in scalp distribution was not statistically reliable. These findings are compatible with the possibility that processing moral violations engages at least some processes that differ from those involved in processing semantic violations. The authors interpret the response to moral violations as reflecting evaluative or affective processing, whereas the response to semantic violations may reflect the updating of the discourse representation. However, these interpretations remain tentative.

Language experience also shapes production and perception in ways that depend on the particular prosodic pattern and the task being performed. Fu et al. (Contribution 5) found that Korean learners’ production of Mandarin disyllabic tone sequences generally improved with proficiency, but the relative difficulty of the sequences remained similar across proficiency levels. Sequences resembling Korean tonal patterns were produced more accurately, whereas sequences ending in Mandarin Tone 2 remained difficult even for advanced learners. Liu et al. (Contribution 7) similarly found that Mandarin listeners’ perception of English interrogative focus varied across tasks. They performed well when identifying prosodic prominence but had greater difficulty identifying focus and judging the naturalness of intonational patterns. Extended experience with English did not eliminate these difficulties. These findings show that the effects of language experience depend on the relationship between the first- and second-language systems and on the aspect of prosody being evaluated.

Hong et al. (Contribution 9) also used ERPs to ask whether the brain forms accent-based groupings across different voices. In an EEG oddball paradigm, native English, L1-Mandarin, and heritage Mandarin listeners heard the word “stopped” produced by 10 Canadian English and 10 Mandarin-accented English talkers. A change to Canadian English elicited mismatch negativity in both the native English and L1-Mandarin groups, even though the standard and deviant tokens came from different talkers. Because the stimuli varied across talkers, this response is consistent with listeners detecting an accent-level regularity despite talker-specific acoustic variation. The heritage Mandarin group did not show a reliable mismatch–negativity response to either accent, although responses within this group varied with Chinese proficiency. The results suggest that pre-attentive responses to accent variation depend on the listener’s language history.

Leung et al. (Contribution 6) address the longstanding question of whether and how speech perception and production are related. Earlier studies by Leung and Wang [3,4] found that Mandarin tone perception and production were linked through F0 slope, the critical perceptual cue for tone categorization, but not through F0 mean. The current study extends this work to second-language learning by examining Indonesian learners of Mandarin over four to six weeks of continued instruction. Across visits, learners gave greater perceptual weight to F0 slope, and their productions became more accurately classified by F0 slope. F0 mean showed a learning effect only in production. Although perception and production changed in parallel for F0 slope, the size of the gains was not correlated across learners. The relationship between perception and production therefore appears to be cue-specific rather than a general correspondence between overall perceptual and production accuracy.

The studies in this Special Issue provide several new insights. First, Fishero et al. (Contribution 1) raise the question of why F0 has a stronger effect than speaking rate when both cues are available. The authors propose that F0 receives greater weight because it more reliably signals talker identity, whereas speaking rate can vary considerably within a speaker. Fishero et al. (Contribution 1) adjusted the target and masker to F0 values of either 175 or 225 Hz. Testing intermediate F0 differences could determine whether the contribution of speaking rate decreases gradually as F0 becomes more distinct or changes once the F0 difference reaches a perceptual threshold. The effect may also depend on how much information remains recoverable from the signal. Ma et al. [5] found that visual information contributed most to word recognition at an intermediate level of auditory noise rather than at the poorest SNR. Following their Bayesian account, speaking rate may contribute most when F0 provides uncertain evidence for segregation but the signal remains sufficiently clear for speaking-rate differences to be detected. Manipulating F0 separation and SNR together would allow this possibility to be tested.

This question is also relevant to cochlear-implant users, who often have limited access to F0 information and difficulty understanding speech under competing-talker conditions. Some cochlear-implant users benefit from voice-gender differences between target and masker speech, although this benefit varies with their ability to keep voices differing in pitch perceptually separate [6]. Given their limited access to F0, an important question is whether cochlear-implant users can rely more heavily on speaking-rate differences to segregate competing speech. Although the effects of F0 and voice-gender differences on speech segregation have been studied in cochlear-implant users [6], it remains unclear whether speaking rate can serve as an alternative segregation cue when F0 is poorly resolved. Comparing listeners with normal hearing and cochlear-implant users while manipulating F0 and speaking-rate differences would test this possibility.

Second, Hong et al. (Contribution 9) provide new evidence that pre-attentive neural responses to accent changes across different talkers vary across listener populations. Native English and L1-Mandarin listeners showed a reliable mismatch response when Canadian English appeared as the deviant accent. Heritage Mandarin listeners did not show a reliable mismatch response to either Canadian English or Mandarin-accented English, and their responses varied with self-reported Chinese proficiency. Their pattern therefore cannot be described simply as intermediate between those of the other two groups. Previous studies have shown that heritage speakers may maintain distinctions or weight phonetic information in ways that differ from both monolingual speakers and late second-language learners [7,8]. Hong et al. (Contribution 9) extend this work by showing that heritage-language experience may also affect how listeners represent accent variation across talkers.

However, the study does not identify the cues underlying these group differences. Listeners may rely on different acoustic or phonological properties when responding to variation in accent across talkers. Future studies could manipulate individual accent cues and measure language experience continuously to determine how these cues contribute to pre-attentive accent differentiation in native, second-language, and heritage listeners.

Third, Meng et al. (Contribution 11) address whether moral violations are processed identically to conflicts with situational or semantic knowledge. Their findings suggest that they are not processed identically. Because the critical sentence and word were held constant across conditions, the different ERP responses cannot be attributed to differences in the target expression itself. A morally unacceptable but physically possible action elicited a response that differed in amplitude and timing from the response to an action that was implausible in the described situation. In addition, moral acceptability explained the posterior positivity beyond what could be explained by plausibility. The moral and semantic conditions therefore elicited different late ERP responses under this manipulation, consistent with the possibility that they engage partially different processes. 

Fourth, Oganyan et al. (Contribution 2) contribute to the debate over whether responses to unfamiliar consonant sequences reflect abstract sonority-based constraints or perceptual pressures. Using eye movements to examine the real-time processing of unfamiliar consonant clusters, they found that listeners’ responses were better predicted by consonant salience than by the sonority hierarchy. This provides new online evidence for the cue-recoverability account proposed by Wright [9]. More generally, the findings caution against treating systematic responses to unfamiliar sound patterns as evidence for abstract sonority-based constraints when those responses may be explained by the perceptual properties of the signal. Cross-language studies could determine when such responses reflect general auditory pressures and when they reflect phonological patterns learned through experience.

Fifth, Tremblay and Kim (Contribution 8) show that bilingual cue weighting cannot be understood as a fixed transfer of L1 perceptual routines to the L2. Spanish-dominant bilinguals relied more on pitch and duration when perceiving English lexical stress, whereas greater English dominance was associated with increased reliance on vowel quality and reduced reliance on pitch and duration. Cue weighting therefore changed with the listeners’ relative experience in the two languages. This finding can be considered within the cue-ecology account proposed by Tremblay et al. [10]. This account differs from traditional approaches that characterize L1 influence primarily in terms of whether the L1 contains a particular phonological category. Instead, it proposes that listeners transfer dimension-specific attentional priorities shaped by how acoustic cues function across the L1 system. These priorities may support L2 perception when the same dimensions provide useful information in both languages, but they may also persist when they are not optimal for the L2. The findings of Tremblay and Kim show that these L1-shaped priorities vary systematically with language dominance, consistent with the possibility that they are recalibrated as relative language experience changes.

Finally, Hromatko and Tadinac (Contribution 12) show that variation associated with the menstrual-cycle phase is specific to the verbal task being performed. Women responded faster in the semantic decision task and produced longer sentences in the verbal fluency task during the mid-luteal phase than during the early follicular phase. These behavioral differences were accompanied by changes in hemispheric laterality. In contrast, neither verbal-reasoning performance nor laterality differed across cycle phases. These findings suggest that the relationship between the menstrual-cycle phase and the verbal processing is task-dependent rather than uniform across verbal abilities. Because hormone levels were not measured directly, however, the observed differences should be attributed to the cycle phase rather than to particular hormonal mechanisms.

The studies in this Special Issue also point to several broader directions for future research. One is to extend findings obtained from adults without diagnosed sensory, neurological, or developmental conditions to clinical populations. This would do more than establish whether an effect can be replicated in another group. It could show which aspects of language processing depend on access to particular sensory cues and which can be supported by alternative information. Research with cochlear-implant users, for example, could reveal how cues are reweighted when pitch information is poorly resolved. The protocol presented by Zuo et al. (Contribution 13) similarly illustrates how studies of neurodevelopmental populations may clarify the processes underlying phonological-feature perception. Comparisons across populations can therefore contribute to theories of language processing as well as to clinical assessment and intervention.

A second direction concerns the influence of social information on speech perception. Listeners’ beliefs about a speaker’s language background, accent, or social identity can affect how the speech signal is interpreted [11]. What remains less clear is how those beliefs are formed in the first place. A listener may draw on the speaker’s voice, pronunciation, appearance, linguistic choices, or the surrounding context, and these sources of information may not always agree. Future research could examine how listeners combine these cues to infer who a speaker is, how quickly that inference is revised, and how it changes the interpretation of subsequent speech. This would extend the study of cue weighting from acoustic information within the signal to information about the person believed to have produced it.

Another important direction is to study language processing during interaction rather than only as an individual response to a fixed stimulus. Although interaction with artificial agents is not directly examined in this Special Issue, this direction provides a prospective extension of its broader themes. In human–human interaction, speakers adapt to one another at lexical, semantic, and prosodic levels. These forms of alignment need not always occur together or make the same contribution to communicative success [12]. Future studies could determine which forms of alignment support successful communication and whether observers can detect that coordination and predict how well a dyad will perform. What is learned from human–human interaction can then provide a basis for studying human–AI and human–robot communication, including whether the forms of alignment that support communication between people also support communication with artificial partners. Finally, moral processing provides a related prospective extension. The same action may be evaluated differently when it is attributed to a person, an AI system, or a robot [13]. These judgments may depend on whether the artificial agent is believed to have knowledge, intention, control, or responsibility. Building on the approach used by Meng et al. (Contribution 11), future studies could hold the described action constant while varying the identity and presumed agency of the actor. This would show how beliefs about an artificial agent affect the interpretation and moral evaluation of its actions. AI and robots could also serve as controlled partners for examining how lexical content, prosody, and feedback influence mutual adaptation, coordination, trust, and judgments of responsibility.
